# Direct access CT for suspicion of brain tumour: an analysis of referral pathways in a population-based patient group

**DOI:** 10.1186/s12875-019-1003-y

**Published:** 2019-08-20

**Authors:** K. Zienius, Ip Chak-Lam, J. Park, M. Ozawa, W. Hamilton, D. Weller, D. Summers, L. Porteous, S. Mohiuddin, E. Keeney, W. Hollingworth, Y. Ben-Shlomo, R. Grant, P. M. Brennan

**Affiliations:** 1 Translational Neurosurgery, Centre for Clinical Brain Sciences, University of Edinburgh, Edinburgh, UK; 2 University of Edinburgh Medical School, Edinburgh, UK; 3 Population Health Sciences, Bristol Medical School, University of Bristol, Bristol, UK; 4 College of Medicine and Health, University of Exeter, Exeter, UK; 5 Usher Institute of Population Health Sciences and Informatics, University of Edinburgh, Edinburgh, UK; 6 Department of Neuroradiology, NHS Lothian, Western General Hospital, Edinburgh, UK; 7 North Berwick Group Practice, North Berwick, East Lothian UK; 8 Department of Clinical Neurosciences, NHS Lothian, Western General Hospital, Edinburgh, UK

**Keywords:** Brain tumor, Brain cancer, Early diagnosis of cancer, Primary health care, General practice, CT scan

## Abstract

**Background:**

Brain tumour patients see their primary care doctor on average three or more times before diagnosis, so there may be an opportunity to identify ‘at risk’ patients earlier. Suspecting a brain tumour diagnosis is difficult because brain tumour-related symptoms are typically non-specific.

**Methods:**

We explored the predictive value of referral guidelines (Kernick and NICE 2005) for brain imaging where a tumour is suspected, in a population-based patient group referred for direct access CT of the head. A consensus panel reviewed whether non-tumour findings were clinically important or whether further investigation was necessary.

**Results:**

Over a 5-year period, 3257 head scans were performed; 318 scans were excluded according to pre-specified criteria. 53 patients (1.8%) were reported to have intracranial tumours, of which 42 were significant (diagnostic yield of 1.43%). There were no false negative CT scans for tumour. With symptom-based referral guidelines primary care doctors can identify patients with a 3% positive predictive value (PPV). 559 patients had non-tumour findings, 31% of which were deemed clinically significant. In 34% of these 559 patients, referral for further imaging and/or specialist assessment from primary care was still thought warranted.

**Conclusion:**

Existing referral guidelines are insufficient to stratify patients adequately based on their symptoms, according to the likelihood that a tumour will be found on brain imaging. Identification of non-tumour findings may be significant for patients and earlier specialist input into interpretation of these images may be beneficial. Improving guidelines to better identify patients at risk of a brain tumour should be a priority, to improve speed of diagnosis, and reduce unnecessary imaging and costs. Future guidelines may incorporate groups of symptoms, clinical signs and tests to improve the predictive value.

## Background

People with a brain tumour see their primary care doctor on average 3 or more times before diagnosis [15], so there may be an opportunity to identify ‘at risk’ patients earlier. Patients with alarming ‘high-risk’ symptoms such as focal neurological deficit caused by a brain tumour are usually diagnosed quickly, either through an emergency department or via urgent referral to a specialist clinic (e.g. TIA/stroke clinic). By contrast, patients with more common and lower-risk symptoms (such as headache, cognitive or personality changes, or a combination of these) are most diagnostically challenging and may experience their symptoms for several weeks before specialist referral [21]. The rarity of this diagnosis means possible cases will usually have another less serious cause.

In the UK, only 1% of adult brain tumours are diagnosed through a “suspicion of cancer” secondary care pathway requiring patients to be seen within 2 weeks of referral [7]. This is one of the lowest of all cancers and is not improving. More adult brain tumours (35%) are diagnosed through routine secondary care outpatient referral pathways [7]. In all likelihood the symptoms in the majority of these brain tumour patients were insufficient to prompt an urgent cancer referral request.

Guidelines exist to support primary care doctors in identification of patients at risk of having a brain tumour to guide who should be prioritised for urgent brain imaging. Kernick and colleagues’ symptom-based guidelines [12] utilise a flag (red/orange/yellow) system that reflects three levels of risk for brain tumour, with emphasis on headache presentations. The National Institute of Health and Care Excellence, (NICE), also developed referral guidelines for adults with suspected CNS cancer. The 2005 [19] guidance was based on groups of symptoms that should precipitate urgent specialist outpatient assessment, typically within 2 weeks. In 2015 [20] the guidelines were simplified to recommend direct access MRI scan within 2 weeks in adults with progressive, sub-acute loss of central neurological function. The 2015 guideline did not provide guidance for patients with other (e.g. cognition, headache) symptoms.

Our objective was to explore the predictive value of the Kernick and NICE 2005 referral guidelines. To do this we investigated the predictive ability of these current referral guidelines to identify patients with a brain tumour when applied to a population-based patient group referred for direct access CT of the head from primary care. We wanted to determine if there was scope for current referral guidelines to be optimised to improve identification of patients in primary care most likely to have a brain tumour based on their symptoms, in order to expedite urgent brain imaging.

## Methods

The Lothian region of South East Scotland serves a population of 840,000. Since 1999 primary care doctors in Lothian have had direct access to outpatient computed tomography (CT) brain imaging via a single referral pathway, for the exclusion of significant intracranial pathology. This is a group of patients whom the referring primary care doctor did not consider needed urgent brain imaging.

**Fig. 1:**
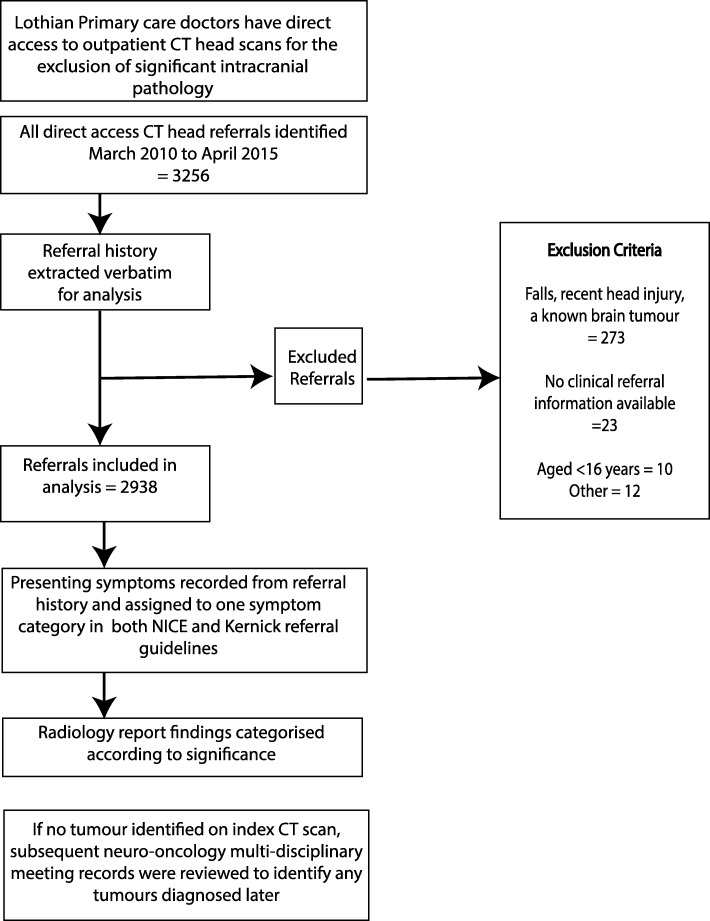
Flow chart of identification and analysis of included referrals

We identified patients aged 16 years or older referred for direct access CT (DACT) between 31 March 2010 and 1 April 2015 from a local electronic database (Fig. 1). All information provided by primary care doctors on the DACT referral was extracted verbatim; the referral is non-structured and there is no minimum dataset. We excluded patients referred for scanning after head trauma to rule out haematoma, those patients with known brain tumour receiving surveillance scans, and where there was no clinical information on the referral form or other electronic sources. All CT head scans were performed in the neuroradiological department at the Department of Clinical Neurosciences, Western General Hospital, Edinburgh, Scotland and reported by a Consultant Neuro-radiologist.

The Kernick referral criteria (Table 1) present 3 symptom groups, where red flag symptoms indicate the probability of an underlying tumour is ≥1%, orange flag symptoms that the probability is between 0.1 and 1%, and yellow flag symptoms indicate a probability of less than 0.1%, but above the population rate of 0.01%. The 2005 NICE referral guidelines included details of symptoms that should prompt urgent referral, consideration of urgent referral, or non-urgent referral (Table 2). In these guidelines we anticipated that ‘headache of raised intracranial pressure’ from the “refer urgently” category may not be associated with an equivalent risk of a brain tumour to ‘symptoms related to the CNS’ and, therefore, we subdivided these into separate categories (Table 2). This allowed a more accurate comparison of symptom complexes at risk for brain tumour.

**Table 1:** “Kernick’s” primary care guidance for imaging patients with suspected brain tumour

Red flag symptoms	
▪ Papilloedema	
▪ significant alterations in consciousness, memory, confusion, or coordination	
▪ new epileptic seizure	
▪ new onset cluster headache	
▪ headache with a history of cancer elsewhere particularly breast and lung	
▪ headache with abnormal findings on neurological examination or other neurological symptoms	
Orange flag symptoms	
▪ new headache where a diagnostic pattern has not emerged after 8 weeks from presentation	
▪ headache aggravated with exertion or Valsalva-like manoeuvre	
▪ headache associated with vomiting	
▪ headache that has been present for some time but have changed significantly, particularly a rapid increase in frequency	
▪ new headache in patient over 50 years	
▪ headache that wake the patient from sleep	
▪ confusion	
Yellow flag symptoms	
▪ diagnosis of migraine or tension-type headache	
▪ weakness or motor loss	
▪ memory loss	
▪ personality change

**Table 2:** NICE 2005 guidelines on specialist referral

Refer urgently	Symptoms related to the CNS, including
	▪ progressive neurological deficit
	▪ new-onset/suspected recent onset seizures
	▪ headaches
	▪ mental changes
	▪ cranial nerve palsy
	▪ unilateral sensorineural deafness
	in whom a brain tumour is suspected
	Headache of recent onset accompanied by features suggestive of raised intracranial pressure, for example:
	▪ vomiting
	▪ drowsiness
	▪ posture-related headache
	▪ pulse-synchronous tinnitus
	▪ or by other focal or non-focal neurological symptoms, for example blackout, change in personality or memory
	▪ a new, qualitatively different, unexplained headache that becomes progressively severe
Consider urgent referral	Patients with rapid progression of:
	▪ sub-acute focal neurological deficit
	▪ unexplained cognitive impairment, behavioural disturbance or slowness, or a combination of these
	▪ personality changes confirmed by a witness and for which there is no reasonable explanation even in the absence of other symptoms and signs of a brain tumour
Consider non-urgent referral	Patients with:
	▪ unexplained headaches of recent onset: either present for at least 1 month, or not accompanied by features suggestive of raised ICP

Presenting symptoms indicated by the primary care referral were individually recorded and assigned to one of these symptom categories in both the NICE and Kernick referral guidelines independently by a junior doctor and a senior medical student (KZ and CI). A Lothian DACT referral is an open text referral through an electronic platform. The referring primary care doctor can write as little or as much information as they feel is necessary. If a symptom appeared in more than one category of the referral guidelines, and the information provided about the presenting symptom was inadequate to assess its severity or characteristics, it was ascribed to the lowest possible category of urgency in the referral guidelines. For example, in the case of ‘headache’ symptom with no qualifying information, the symptom would be recorded as the non-urgent category in the NICE referral guidelines.

If there was disagreement as to which referral guideline category the presenting symptom should be ascribed to, one of the other authors (PB), a consultant neurosurgeon, made the final judgement. The radiological interval was determined as the time from the date of referral for DACT to the date of brain imaging.

Radiology reports for each patient were categorised by KZ into one of three groups according to the findings reported by the consultant radiologist: 1) abnormal significant - intracranial tumour 2) abnormal significant - non-tumour and 3) normal/non-significant incidental finding. All scan reports where the imaging was not reported as ‘normal’ were reviewed by a consultant neurosurgeon, (PB), to assess whether the abnormality identified was likely to be significant in the context of the referral history. This assessment was based on the likely diagnosis (e.g. tumour), the size and location of the abnormality, and/or whether the abnormality was thought likely to be responsible for the presenting symptoms, considering any opinion reported by the consultant neuroradiologist. Where necessary, the images were reviewed. Findings were considered ‘significant’ if clinical details correlated with imaging findings, or if the imaging findings alone were suggestive of requiring further investigations irrespective of the symptoms. Findings were considered ‘non-significant’ if they were reported as within normal limits for age (e.g. some degree of atrophy or small vessel disease) or were not related to the presenting symptom(s) recorded on the referral.

For all patients where a tumour was not diagnosed on imaging, we determined whether within the duration of the study there was evidence of a tumour diagnosis. We did this by examining all neuro-oncology multidisciplinary meeting (MDM) minutes for the same 5-year time-period at Edinburgh Centre for Neuro-Oncology (ECNO), and any subsequent imaging reports on the patient’s electronic health record, for a minimum of three years.

Confirmation of CNS tumour diagnosis was based on histopathology for all significant tumours.

We assessed how the imaging report might have influenced the referring primary care doctor. Three practising primary care doctors, (WHa, based in England, LP and DW based in Scotland) reviewed ‘abnormal’ imaging reports alongside the accompanying referral text and determined a potential action plan for follow-up according to the classification system in Table 3. We examined the management plans according to whether the symptom met the NICE 2005 guidelines for urgent or non-urgent referral.

**Table 3:** Potential action plan suggested by GPs based on direct access head imaging

1	Normal - does NOT explain the presenting symptom(s)
2	Abnormal, but not requiring further investigation - explains the presenting symptom(s) and can be managed in primary care
3	Abnormal, but not requiring further investigation - does NOT explain the presenting complaint, but can be managed in primary care
4	Abnormal, requiring further investigation (such as follow up scans or referral), but NO suspicion of tumour
5	Tumour suspected, requiring referral/follow-up

### Statistical methods

To examine the diagnostic performance of the imaging/referral guidelines we calculated the diagnostic odds ratios using a logistic regression model for the presence of CNS tumour. Gender and age were considered as confounding variables and were adjusted for in the logistic regression model. Correlation between NICE 2005 and Kernick’s referral guideline symptom categories was analysed using the weighted kappa statistic. A frequency table was created of the ‘abnormal’ findings reported, and of the action plan determined by the primary care doctor. The level of statistical significance was set at *p* < 0.05. The data were analysed using Stata/MP 14.2.

## Results

Over a 5-year period, 3256 head scans were performed. After excluding 318 scans according to pre-specified criteria (see Fig. 1 flowchart), 2938 records were reviewed. The mean age was 55.6 years (SD = 18.56) and there were more females (1748, 60%); Table 4.

**Table 4:** Baseline demographics for patients investigated with direct access CT head imaging over 5-year period (*N* = 2938)

Patient variable		Significant Brain tumour
	All	Present (*n* = 42)
Gender		
Female, N (%)	1748 (60)	23 (55)
Age (years)		
Mean, SD	55.6 (18.56)	60.2 (14.38)
Age group in years, N (%)		
16–29	335 (11.4)	1 (2.4)
30–39	287 (9.8)	1 (2.4)
40–49	436 (14.8)	9 (21.4)
50–59	547 (18.6)	6 (14.3)
60–69	567 (19.3)	15 (35.7)
70–79	464 (15.8)	7 (16.6)
80+	302 (10.3)	3 (7.1)
Radiological interval (days)		
Mean [95% CI]	17.3 [16.9,17.6]	14.6 [12.4,16.7]*

*statistically significant difference (t-test), *p* = 0.05

Assessors agreed as to categorisation of the presenting symptom in 97% of referrals. From 2938 scans, 53 patients (1.8%) had intracranial tumours reported, of which 42 were significant (diagnostic yield of 1.43%). Eleven tumours were thought to be incidental, unrelated to symptom(s) precipitating referral, and likely to be benign meningiomas based on the radiological appearance. None of these patients proceeded to surgery within the time course of the study. No patient without intracranial tumour on the DACT imaging report was subsequently identified to have a tumour on imaging within the study.

Of the significant tumours, 17/42 (40%) patients had metastases, 8/42 (19%) pituitary tumours, 7/42 (17%) meningiomas, 5/42 (12%) glioblastoma multiforme (GBM), 4/42 (10%) non-GBM glioma, and 1/42 (2%) a CNS lymphoma.

Brain tumours occurred in each of the Kernick categories with the expected frequencies: red flag 3.7% (expected > 1%), orange flag 0.7% (expected 0.1 to 1%) and yellow flag 0.09% (expected 0.01 to < 0.1%) (Table 5). Kernick’s red-flag group was the only one with a significantly increased risk of brain tumour (OR 5.73, 95% CI 2.21–14.84) (Table 5). Using the NICE 2005 referral guidelines, ‘symptoms related to CNS’ had significantly elevated odds ratio for presence of a brain tumour (OR 5.21, 95% CI 1.81–14.92) compared to the symptoms recommended for non-urgent referral (Table 6).

**Table 5:** Frequency of tumours from direct access CT grouped based on Kernick referral criteria. Odds ratio with 95% CI for each flag symptom, for the presence of a brain tumour

		Brain tumour						
	Total	Absent		Present				
		Freq	%	Freq	%	Odds ratio	95% CI	PPV
Red flag symptoms	1141	1109	92	32	3.7	**5.73**	**2.21–14.84**	2.8
Orange flag symptoms	728	723	99	5	0.7	1.34	0.38–4.68	0.7
Yellow flag symptoms	1069	1064	99	1	0.09	reference		0.5

Adjusted for age and sex. An odds ratio of 1.0 indicates the odds of exposure among case-patients are the same as, or similar to, the odds of exposure among controls

**Table 6:** Frequency of referrals for direct access CT grouped based on clinical criteria from NICE 2005 guidelines. Odds ratio with 95%CI/PPV for each criteria of NICE 2005 guidelines for the presence of a brain tumour

		Brain tumour						
	Total	Absent		Present				
		Freq	%	Freq	%	Odds ratio	95% CI	PPV
Symptoms related to the CNS	1150	1117	97	33	3.0	**5.21**	**1.81–14.92**	2.9
Headache & features of raised ICP	473	469	99	4	1	1.65	0.41–6.67	0.8
Sub-acute deficits focal/personality/cognitive/behavioural	525	524	99	1	< 1	0.31	0.03–2.79	0.2
Simple headache	790	786	99	4	1	reference		0.5

Adjusted for age and sex. An odds ratio of 1.0 indicates the odds of exposure among case-patients are the same as, or similar to, the odds of exposure among controls

If the tumours we deemed incidental were instead included in the analysis, the OR for red flag symptoms reduced to 3.86 (1.78–8.38) and for orange symptoms increased to 1.46 (0.56–3.85). For the NICE 2005 categories, the odds ratios changed to 3.59 (1.5–8.7), 1.9 (0.63–5.69), and 0.54 (0.13–2.2) for CNS, raised ICP and subacute groups, respectively.

### Frequency of non-tumour radiological diagnosis

Five hundred fifty-nine (19.2%) scans reported a non-tumour finding (Table 7). 177 (31.7%) of these findings were considered significant based on patient symptoms and radiological opinion. Cerebral atrophy (45.8%), vascular abnormalities (27.1%) and sinus disease (13.6%) were the most common significant findings.

**Table 7:** Frequency of non-tumour findings on direct access CT imaging

Abnormality	Frequency	
	Total	Significant (%)
Cerebral atrophy	206	81 (39.3)
Vascular	195	48 (24.6)
Sinus disease	58	24 (48.0)
Arnold Chiari malformation type 1/tonsillar ectopia	27	9 (33.3)
Benign cystic lesion	20	2 (10.0)
Ventricular abnormality	11	8 (72.7)
ENT tumour	4	1 (25.0)
Other	38	4 (10.5)
Total	559	177 (31.1)

We examined how well the Kernick and NICE 2005 referral guidelines performed at identifying all significant abnormalities, including brain tumours. The odds ratio for red flag symptoms was 0.7 (0.5–0.9) and for orange symptoms 0.38 (0.25–0.58). For the NICE 2005 categories, the odds ratios changed to 2.4 (1.5–3.9), 1.57 (0.85–2.9), and 4.76 (2.88–7.87) for CNS, raised ICP and subacute groups, respectively. This indicates a worse performance for all categories except for subacute symptoms in the NICE referral guidelines, when compared with the performance for identifying only brain tumours.

### Management of radiological findings in primary care

We determined possible management of the 559 patients with non-tumour findings in primary care from the available referral history and CT brain imaging findings (Table 8).

**Table 8:** Frequency table action plan by primary care doctor for reported non-tumour findings from direct access head imaging, N (%)

	Normal - does NOT explain the presenting symptom(s)	Abnormal, but not requiring further investigation - explains the presenting symptom(s) and can be managed in primary care	Abnormal, but not requiring further investigation - does NOT explain the presenting complaint, but can be managed in primary care	Abnormal, requiring further investigation (such as follow up scans or referral), but NO suspicion of tumour	Tumour suspected, requiring referral/follow-up
Cerebral atrophy	12 (5.8)	53 (25.7)	76 (36.9)	65 (31.6)	0
Ventricular abnormality	1 (9.8)	1 (9.8)	5 (45.5)	4 (36.4)	0
Benign cystic lesion	0	2 (10)	6 (30)	12 (60)	0
Sinus disease	7 (12.1)	9 (15.5)	23 (39.7)	19 (32.8)	0
Arnold Chiari malformation type 1/tonsillar ectopia	5 (18.5)	2 (7.4)	12 (44.4)	8 (29.6)	0
ENT lesion	0	0	3 (75)	1 (25)	0
Vascular	12 (6.2)	37 (19)	74 (37.9)	72 (36.9)	0
Other	7 (18.4)	2 (5.3)	15 (39.5)	14 (36.8)	0
Total	44 (7.7)	106 (18.6)	219 (38.4)	195 (34.2)	6 (1.1)
NICE category: Urgent referral (N, %)	35 (6.5)	97 (18)	195 (36.2)	172 (32)	39 (7.2)
NICE category: Non-Urgent (N, %)	9 (12)	9 (12)	24 (32)	29 (38.7)	4 (5.3)

18.6% of imaging abnormalities were determined to require no further follow-up but did explain the patient’s presenting symptom. 38.4% of radiologically reported abnormalities did not explain the patient’s presenting symptoms, but care could be managed in primary care. 34.2% of patients required referral for further imaging or specialist assessment. 38.7% of referrals with an imaging abnormality that met the NICE 2005 ‘non-urgent classification’ would still have been referred for further imaging or specialist opinion.

We performed a cross tabulation to compare the three principal categories of NICE and Kernick referral guidelines based on recommended speed of referral (Table 9). Whilst 100% of Kernick red flag cases were also classified as refer urgently according to the NICE 2005 guidance, there was overall quite a lot of disagreement and the weighted kappa showed only modest agreement beyond chance (0.33). This highlights that the different referral guidelines are making different management recommendations for any given symptom.

**Table 9:** Cross Tabulation of symptoms within Kernick and NICE 2005 referral guidelines based on recommended speed of referral

			NICE 2005 categories			
			Refer Urgently	Consider urgent referral	Non-urgent referral	Total
Kernick	Red Flag	Count	1141	0	0	1141
		% within Kernick	100.0	0.0	0.0	100.0
		% within NICE	70.3	0.0	0.0	38.8
		% of total	38.8	0.0	0.0	38.8
	Orange Flag	Count	468	4	256	728
		% within Kernick	64.3	0.5	35.2	100.0
		% within NICE	28.8	0.8	32.4	24.8
		% of total	15.9	0.1	8.7	24.8
	Yellow Flag	Count	14	521	534	1069
		% within Kernick	1.3	48.7	50.0	100.0
		% within NICE	0.9	99.2	67.6	36.4
		% of total	0.5	17.7	18.2	36.4
Total		Count	1623	525	790	2938
		% within Kernick	55.2	17.9	26.9	100.0
		% within NICE	100.0	100.0	100.0	100.0
		% of total	55.2	17.9	26.9	100.0

## Discussion

### Summary

Our study is the first to examine the diagnostic utility of both the NICE 2005^19^ and Kernick [12] guidelines in a population-based patient group and to consider the relevance of significant non-tumour pathology.

The availability of direct primary care access to brain imaging (DACT) varies across the UK. In areas where it is available the Kernick and NICE guidelines may be used to inform referral decisions. Broadly, based on symptoms (red flags or NICE 2005 ‘symptoms related to the CNS’) primary care doctors can identify patients who have a brain tumour with a 3% PPV. Nearly 80% of patients whose CT revealed a brain tumour had ‘symptoms related to the CNS’ on NICE 2005 or Kernick’s red-flag symptoms. Overall, the Kernick system performed as predicted [12].

Nineteen percent of scans reported non-tumour findings, 18.6% of these non-tumour findings were thought by primary care doctors to explain the patient’s symptoms. One third of patients with a non-tumour abnormal finding were judged to require onward specialist referral. Access to DACT will influence management of more patients who do not have brain tumours than those that do. Most significant non-tumour findings fall into Kernick’s yellow flag group, and into the sub-acute focal deficits of NICE, so DACT should not be restricted to the ‘refer urgently’ and red-flag symptom categories. Further, whether patients with lower prevalence symptoms (e.g. yellow and orange flags) might also benefit through faster ruling in or out of cancer and the diagnosis of other pathology, and whether this is justified by the cost and potential harm (radiation and over diagnosis) of CT, requires further study.

Whilst existing referral guidelines may meet their intended threshold of specificity, these guidelines are insufficient to adequately stratify patients. Moreover, the different referral guidelines make different management recommendations in terms of urgency for some symptoms. For example, all of the Kernick red flags are also in NICE refer urgently, but 30% of NICE refer urgently are either in Orange and even a few in Yellow flags. These referral guidelines are recommending quite different decisions for some patients, reflecting the evidence that any individual patient symptom is poorly predictive of a brain tumour [21]. This is important, because inefficient guidelines crowd the referral system, adding to delay in diagnosis for those patients who do have a brain tumour. Whether more liberal DACT criteria is a good use of NHS money requires further research.

### Strengths and limitations

This is the largest reported population-based study of primary care imaging referrals for suspected intracranial abnormality. Grouping was performed by two authors, and a third where necessary, to increase accuracy. All scans were performed at a single clinical neuro-sciences centre and were reported by a consultant neuro-radiologist. As this was part of a retrospective study it allowed sufficient follow-up to record false negatives for a brain tumour with respect to the initial diagnostic CT scan; we did not identify any. A strength is that we examined scan utility in patients with non-tumour pathology, based on a consensus opinion. Despite the large number of referrals that yield of brain tumour cases was not large, hence we have imprecise estimates for our odds ratios.

Many patients with sudden onset symptoms that precipitate immediate hospital assessment at an emergency department will also fall into the Kernick red flag category. Our approach therefore underestimates the predictive ability of the Kernick system in the whole brain tumour population, although our data does reflect the situation in primary care when uncertainty about a brain tumour diagnosis exists.

### Implications for practice and research

The non-specificity of many symptoms associated with brain tumours makes it difficult to identify patients to prioritise for brain imaging. Our data demonstrates that existing guidelines provide some assistance to the primary care doctor. We utilised the 2005 NICE guidelines because they provided a greater granularity of patient symptoms than 2015 guidelines. Since we demonstrated that only ‘symptoms related to CNS’ is a useful predictor of whether or not a patient may have a brain tumour, the simplification in 2015 may be justified.

A relatively large proportion of our patients were referred for headache only (26.9%). Certain headache features may raise suspicion of an underlying brain tumour [2], but are still not specific [22]. We previously demonstrated that patients with non-specific symptoms such headache, cognitive changes or personality changes are subjected to the longest delay from onset of symptoms to brain tumour diagnosis [21]. Combining these symptoms may help identify patients to prioritise for brain imaging [21], but this must be balanced against the ‘risk’ of detecting pathology incidental to the clinical presentation.

In our cohort, there were no false-negative results.

The NICE 2015 guidelines recommend cranial MRI for suspected brain tumour in patients with progressive sub-acute loss of central neurological function (CT only if MRI contra-indicated). The low uptake of imaging for brain tumours in primary care compared to other cancers [6] may in part reflect variation in access to brain imaging. There are fewer MRI scanners in the UK than almost any other Western European country [16]. Our data suggest CT imaging may be adequate as a test in patients deemed at risk of a brain tumour based on headache symptoms. CT is the standard initial imaging modality for patients with acute onset focal stroke-like symptoms [5] and is not diagnostically inferior in identifying clinically significant pathologies for non-acute headache presentation [8, 11]. Cranial CT costs less and is more readily available than MRI. MRI might be reserved for specific patients, such as those with new onset seizures, where a radiologically subtle low-grade tumour is more likely [26]. Potential risks of ionising radiation are sometimes cited to recommend MRI over CT, although an individual patient’s risk is small [24]. It is projected that 1 in 8100 women aged 40 years will develop cancer from a single head CT [24]. The risks may be doubled for 20-year-olds [24] so It may be necessary to prioritise MRI in younger patients.

### Comparison with other studies

Our diagnostic yield of the referral guidelines for a significant brain tumour was 1.43%, slightly higher than that reported by Benamore [3] (for any symptom presentation - 1.28%) or Simpson [23] (for symptom of headache only - 0.5%), both with open access CT.

For some patients a negative scan for a brain tumour provides reassurance [1, 10]. Incidental findings are not uncommon, even in asymptomatic patients, and can result in additional patient worry, investigations and cost [3, 4, 11, 18]. Nearly a fifth of all scans in our study identified a non-tumour finding, similar to patients referred for imaging with any symptom [3, 17] or headache only [11]. 18.6% of our non-tumour findings likely explained the symptoms precipitating referral, although the incidence of significant cerebral atrophy may have been overstated, because we performed qualitative estimation of atrophy, which is known to have inter-rater variability [14]. For two thirds of abnormal radiological findings, primary care doctors in our study would be confident to manage patients in primary care, reflecting other reports [3, 11]. In keeping with previous findings [3], CT for ‘simple’ headache (“non-urgent” category according to NICE 2005) may require further follow-up or additional investigation in 38.7% of cases where a non-tumour abnormality is found.

Primary care doctors often report difficulty interpreting neuro-radiological reports, particularly incidental abnormalities [25], and find radiologists’ recommendations for further treatment, referral or non-radiological investigation valuable [9]. There is no agreed consensus among radiologists on reporting of incidental findings and/or recommending follow-ups. Primary care doctors rely on their judgement interpreting and relaying these reports to patients, which may cause anxiety for the primary care doctor and lead to additional referrals [13]. A recent qualitative prospective study with 20 GPs identified a need for standardised reporting of scans to improve GP’s use of direct-access MRI [25], which may also be appropriate for CT scans.

## Conclusions

Guidelines should provide an evidence-base to assist primary care doctors in identifying patients most at risk of having a brain tumour (i.e. diagnostic accuracy), but also the fastest route to achieve diagnosis (e.g. direct access imaging versus urgent secondary care referral). Direct access CT scanning has a low false negative rate. However, existing referral guidelines are insufficient to adequately stratify patients based on their symptoms according to the likelihood that a tumour will be identified on brain imaging. Improving guidelines to better identify patients at risk of a brain tumour should be a priority, to improve speed of diagnosis, and reduce unnecessary imaging and costs. Future guidelines may need to incorporate groups of symptoms, clinical signs and tests to improve the predictive value. In patients referred for DACT, identification of non-tumour findings may be important for patients. Earlier specialist input into interpretation of these non-tumour abnormality images may be warranted and appreciated.

## Data Availability

The dataset used during this study are available from the corresponding author in reasonable request.

## Abbreviations

CNS: Central nervous system
CT: Computed tomography
DACT: Direct access CT
MRI: Magnetic resonance imaging
NICE: National institute of clinical excellence
